# Observations upon the Origin and Latent Period of Fever

**Published:** 1828-01-01

**Authors:** 


*Observations upon the Origin and latent Period of Fever.* By Henry Marsh, M.D. &c. Assistant Physician to Steeven's Hospital, &c. &c. &c. Octavo, pp. 83. Dublin, 1827.

[From the Dublin Hospital Reports, Vol. IV.]

The subject discussed in this paper is of the greatest importance, and has hitherto attracted but comparatively little attention. Great labour has been expended on the investigation of the causes, remote, predisposing, and proximate, of fever; but the interval between the reception or the impression of the cause of disease, and its *manifest* operation on the animal economy, in the shape of unequivocal disorder, seems to have excited little examination, or even speculation. It would be exceedingly curious—perhaps very useful, to know what is going on during this period of incubation—to ascertain where the poison resides—on what organs or structures it is secretly operating—and how the constitution is employed in meeting or resisting the deleterious agent. If these things could be ascertained, we would probably be able to counteract some of the causes of diseases, (as the contagions and miasmata) during their nascent or latent periods of existence in the animal economy.

The paper of Dr. Marsh occupies a considerable space in the last volume of the Dublin Hospital Reports, and we deem it amply sufficient for an article in the present Number of our Journal.

Dr. M. considers, *first*, the manner in which febrile disease is produced—*secondly*, the symptoms of the latent period—*thirdly*, the treatment adapted to *this stage* of the disease.

I. *Mode of Production.* Mere reasoning will not unravel this mystery. Nature herself must be interrogated, and her operations faithfully noted. If this had been done always, instead of theorizing in the closet, he thinks the subject of contagion or infection (he properly considers the two words synonymous, as all infectious miasms must necessarily be contagious before they can operate on the human body) would not now have remained so much in obscurity. The following are the facts, in the order of their occurrence, which drew our author's attention to the subject, and seemed to throw some light on an obscure point of pathology.

1. A female, aged 24 years, admitted into one of Dr. Crampton's wards in the Whitworth Hospital, 24th November, 1817, makes, voluntarily, the following statement:—

"On the Wednesday preceding the day of her admission, a person not yet recovered from fever came into the house where she then was, and sat down close beside her. She became immediately sensible of a heavy disagreeable odour arising from the person of this individual, which disgusted her exceedingly; she was instantly affected with head-ache, and became so weak that with difficulty she could move her limbs or stand. That very evening long continued rigors came on, followed by heat, to which succeeded perspiration; she spent a restless night, slept in an agitated manner, and awoke unrefreshed. She was admitted into the hospital, labouring under the ordinary symptoms of fever in an intense degree; she complained of severe head-ache, great prostration of strength, and was covered with large and florid petechiæ: her fever was tedious, and her recovery slow." 457.

2. On the 17th June, 1818, Nurse Smyth, in the Richmond Penitentiary, administered an enema to a typhous patient, during which operation, the patient's bowels were suddenly evacuated.

"The smell issuing from the fæces produced immediate and most intense head-ache, and her strength at the same time was so completely exhausted, that she had neither power to move nor to support herself on her limbs. In a few hours afterwards she was seized with a severe rigor; but, being placed close to a large fire, she became warm, yet not without occasional sensations of chillness; feeling as if some cold substance moved slowly along her back. Typhous fever, of a severe character, yet presenting no unusual symptoms, supervened. The patient to whom the enema had been administered died in two days afterwards, having exhibited the worst characters of petechial fever." 457.

3. A nurse applied leeches to a patient in the last stage of typhus, whose body was covered with petechiæ. The smell of the patient's breath greatly incommoded the nurse. Shortly afterwards she had a rigor, and felt as if cold water was trickling along the spine. Fever quickly ensued, after taking an antimonial emetic; but it was mild, and she recovered.

4. A nurse was employed in washing the linen of an old woman labouring under typhus, covered with petechiæ, and exhibiting all the worst symptoms of that fever. The smell of the sheets affected the nurse powerfully and disagreeably. She was instantly seized with head-ache and great debility. Soon afterwards she had rigor, and when Dr. Marsh saw her, she had head-ache, nausea, and prostration of strength. Her skin was hot, pulse frequent and full. Venesection—leeches to the temples—free purgation. Her fever subsided on the fifth day.

5. A young gentleman, from motives of humanity, visited some poor people living in a wretched apartment, in one of the filthy lanes of the City, and where two people were labouring under bad fever. On opening the door, the smell immediately sickened him—he returned home unwell—and spent an agitated night. Next day, Dr. M. met him in the street, and was struck with his altered appearance. He was pale—his skin had a dingy appearance—there was a livid circle below the eyes—and he complained of great languor and debility. In the evening, his eyes were deeply suffused—cheeks flushed—skin intensely hot—mouth parched—tongue loaded—pulse 120, full and resisting—breathing hurried—frequent sighing. Temporal arteriotomy to 24 ounces, by which he was much relieved; but had a wretched night, and was delirious. Nevertheless he awoke next morning free from fever, and only complaining of debility.

6. A nurse, while occupied about a patient labouring under petechial fever, (and who died two days afterwards,) was struck with a heavy smell, which affected her disagreeably, and produced head-ache, accompanied by languor of several days duration. She was at length obliged to take to her bed, and, during a protracted fever, never ceased to complain of intense head-ache and præcordial oppression. Her convalescence was tedious, and it was a long time before she got rid of the weight and oppression at the cardiac region.

7. The Rev. Mr. Fletcher visited, before dinner, a small hospital for fever cases among the parochial poor. While speaking to a woman, he found himself standing among straw, in which was some feculent matter voided by a fever-patient. The effluvium struck him forcibly—and he immediately felt pain of head and sickness of stomach, with such excessive debility that he was unable to stand, being obliged to support himself against an adjoining gate-post. He had rigor that same evening, and a fever of unusual severity ensued, during three days of which, the pupils were permanently dilated, and he lay in a state of total insensibility.

8. Dr. Crawford (one of our author's fellow-labourers) gives the following account, in his Thesis, of the manner in which he became affected with the fever.

" 'On entering a ward very early in the morning, where lay a woman labouring under the worst description of fever, as I approached the bed, I perceived a disagreeable fetor, and was instantaneously struck with head-ache and nausea. In two days afterwards I was seized with a fever of the same character.' " 461.

9. In a few hours after visiting a bad case of typhus, Dr. James Clarke met Dr. Cleghorn in the street, and observed that he had got a *fugh* (a word in Scotland meaning a heavy disagreeable smell) at the hospital that day, of which he could not get rid. He dined, however, heartily at Dr. Ferguson's; but not finding himself well in the evening, he took six grains of calomel. Next day he could eat nothing. He now became affected with fever, and died on the eleventh day.

" 10. Dr. Waring, on visiting a patient labouring under typhus fever, found the room so close that he instantly broke a pane of glass in the window. On his return home, he told Mrs. Waring that he had got a knock on the head, which all the College of Physicians could not cure. Dr. Cleghorn saw him on the next day, when he repeated to him the same words. He had been engaged in lecturing for Dr. Cleghorn during the preceding days; and had very much overworked himself. The fever, thus produced, was fatal." 462.

11. Dr. Parkinson visited a fever patient, residing in a small apartment, destitute of ventilation, and, while standing close to the bed, the patient suddenly sat up. "Having perceived a most disagreeable smell, he observed at the instant that he had caught the infection." He rushed to the window and forced out a pane of glass. Next day he made no complaint, but appeared dull and out of spirits—he was flushed, and yet he said he felt cold. On the succeeding day he had severe head-ache, and in a few days more he died.

12. The following was Dr. Marsh's own case. On the 4th February, he found himself in good health and spirits. On the 5th he took a scanty and hurried breakfast, and the business of the day was laborious and fatiguing. At six in the evening he arrived at the hospital, and visited the fever ward, and, on turning down the bed-clothes of a fever patient, he perceived a highly disagreeable odour, by which he was oppressed and overwhelmed. He hastened home, not feeling very well, and ate a hearty dinner; but felt himself so chilly in the evening, that he could not keep himself warm by all the coats and cloaks he put on. He put his feet in hot water that night, which brought out some perspiration. On the 6th, he was unable to leave his bed. On the 7th, he went out in a carriage, perspiring, yet chilly, with severe head-ache, aggravated by coughing or moving—great depression of spirits, amounting to absolute despair—mental agony—copious secretion of limpid urine—clammy perspirations of disagreeable odour—distressing sensation in the stomach, with occasional vomiting—disagreeable taste in the mouth—tongue still clean—pulse scarcely accelerated—no heat of skin. "To a short period of delirium succeeded idiotic manner and gesture—insensibility—spasmodic contraction of limbs—subsultus tendinum." For several hours his situation appeared hopeless; and during convalescence, so much was the nervous system debilitated, and so great was the exhaustion, that frequent fits, accurately resembling hysteria, occurred, excited by unpleasant thoughts, or some slight mental emotion. Upon return of consciousness in this fever, every object appeared to Dr. M. to be black, and beautifully exact and regular in its outline.

A great number of other similar cases are related—many of them happening to medical men themselves; but we think it is unnecessary to adduce additional proofs that a poison is received from the bodies of febrile patients which often produces fever in the visiter.

We shall now proceed to Dr. Marsh's observations on "the manner in which the febrile miasm, and other poisons, act primarily on the living body." He remarks that—

"An opinion is entertained by *many*, (he might have said by *most*,) that poisons, to produce their specific effects, must necessarily be first absorbed and mingled with the circulating fluid; and that, subsequently to this process, their peculiar injurious effects are called into action." 475.

Dr. M. thinks there are strong reasons to doubt the correctness of this opinion. The effects of poison are sometimes too quickly produced, he imagines, to be compatible with the process of absorption. That an instantaneous and very violent impression may be made on the sentient extremities of the nerves, so as to give a great shock to the constitution, or even induce death, there can be no doubt. A dose of prussic acid may unquestionably destroy life by its action on the nerves of the stomach, independently of any absorption. A powerful mental impression has often obliterated at once all the vital functions; and absorption would scarcely be contended for here, by the most stanch materialist. Dr. Marsh having adduced examples of the rapid manner in which narcotic poisons act on the nerves, and through them on the brain, proceeds to apply the same explanation to the miasmal or febrific poisons. But certainly we think his reasonings are not quite convincing.

"From these facts, (those cases and others which have been detailed) it appears, that the poison of contagion produces its effects with the same rapidity as the narcotic poisons, to which we have alluded. Headache, debility, sickness at stomach or vomiting, are amongst the symptoms first perceived; these sensations, with the rapidity of an electric shock, are at the instant produced. This injurious impression upon the sentient extremities of the nerves is, in a few rare instances, so violent as to be very speedily fatal. More frequently the impression is less violent, but sufficiently strong to disturb the health, produce unpleasant sensations, and lay the foundation for disease. By far the greater number of patients, labouring under contagious fever, are not at all aware of the circumstances connected with the origin of their complaint; the impression at the time of their exposure being in general either unheeded or forgotten. Indeed the impression is oftentimes so slight, as to lead one to think that contagion does no more than predispose to fever, and determine the nature of the disease, of which exposure to cold, fatigue, or some such accidental circumstance, is the immediately exciting cause; so that there appears much reason to believe that many are so mildly affected, that, were it not for the super-addition of an exciting cause, they would altogether escape fever; hence it happens that numbers, affected with contagious fever, trace the origin of their complaint exclusively to cold, wet, and other exciting causes of disease; the time and circumstances of exposure to contagion having been entirely forgotten. Cases of this kind, which are by far the most numerous, throw but little light upon the origin of fever. It is only by a careful observation of facts of occasional and rare occurrence, such as those recorded in this paper, in which the effects of contagion are well-marked and striking, that we can hope to obtain certain and satisfactory results. In the instances recorded, in which the patients were sensible at the moment of the impression made upon the system by the febrile effluvia, some of the earliest symptoms complained of were headache, vertigo, nausea, vomiting, a sensation of coldness, and in many instances a sudden and complete prostration of strength. These primary symptoms did not always continue: in some instances they became less severe, or did in a good measure subside; yet the patient was not thereupon restored to health, on the contrary, a permanent injury was inflicted, and the foundation was laid in the system for a series of phenomena, which, taken together, constitute fever. The symptom, which is generally considered to mark the commencement of a febrile movement in the system, is that commotion of the nervous functions which has been technically termed a rigor; the interval of time between the injurious impression of the human miasm or other cause of fever, and the rigor or chillness ushering in fever, is that which has been denominated the latent period. In some instances the infliction of the injury and the first symptoms of fever are simultaneous; in these cases, a latent period can scarcely be said to have existed.

In other instances the fever begins and advances in a manner so slow and gradual, and its symptoms are so mixed up with those which belong to the latent period, that it is difficult to distinguish the one from the other; not infrequently the chillness is so slight, and of such frequent recurrence, and alternating as it sometimes happens with sensations of heat, that it becomes difficult, nay often impossible to ascertain the precise period, at which the fever may be said to have commenced. Indeed it may be observed that those cases of fever in which re-action, or a distinct febrile movement, either does not at all, or does but feebly and imperfectly take place, are amongst the most dangerous and formidable varieties of this disease; but the more ordinary march of nature is, that there shall be an interval of time, variable in duration, between the application of the poisonous effluvia to the surface, and the commencement of the febrile movement. During this interval of time a state of perfect health does not exist. In some instances, the deviation from health is so slight as scarcely to be perceived or regarded; in some it is obvious, and is rendered manifest, not only by the patient's own sensations, but also by the altered expression of his countenance; and there are instances, in which the latent period is one of the greatest danger, and one during which symptoms the most alarming and formidable do occasionally arise. The symptoms which characterise this period, whether they be slight or whether they be severe, indicate a disturbance affecting primarily and peculiarly the nervous system. This state of ill-health is evinced more by disturbed sleep, uneasy dreams, painful forebodings, depression of spirits, mental inquietude, loss of muscular tone and vigour, than by any definite symptom of disease." 485.

The above views are supported by Drs. Barker and Cheyne in their account of the late epidemic in Ireland. They observe:—"We are not of opinion that the time between the exposure to contagion, and the formation of the disease thereby caused, is a period of health: the nervous system was affected previous to any disorder of the circulating system." This position they illustrate by examples.

It has always been our opinion, that the febrific cause made its first impression on the nervous system; but this, we think, does not disprove that absorption *first* takes place—still less does it prove that absorption is not necessary at all. When we reflect that there is no part of the nervous system completely exposed, it is hardly possible to conceive that a poison acts on the sentient extremities of the nerves, without some degree of absorption having previously obtained. It may not be necessary that a poison should reach the brain, or make the round of the circulation before it produces some effects on the animal economy; but still we think that the probability is, that absorption, sooner or later, takes place.

The interval of time, between the infection and the manifestation of fever, deserves particular investigation. The clearest conception of the laws of nature, on this point, may be derived from an analysis of the progress of those exanthematous fevers propagated by inoculation. Thus, the variolous poison is applied to an abraded surface—an interval of apparent inaction elapses—then a local excitement takes place in the spot where the poison was inserted, and a disease similar to that from which the infection was taken, is produced. Although unseen changes are no doubt going on in the part, yet, as they are not cognizable by the senses, we may call this the first latent period. From the formation of the local disease, till the occurrence of rigors, or other symptoms, ushering in the constitutional disease, a second period intervenes. So, in diseases originating from impressions of cold on the surface of the body, a latent period is found to exist, as every one knows, though a narrow observance of phenomena would probably detail some obscure constitutional disturbances, which usually pass unobserved.

"I know not any conditions of disease more strikingly illustrative of these principles, than the effects immediate and remote of protracted operations, severe injuries, and extensive burns. In all of these a strong and injurious impression is made upon the nervous system; a period of disturbed nervous function, one corresponding with the latent period of fever, ensues. In severe injuries this is a time of the utmost danger; the injury inflicted upon the nervous system being oftentimes so great, that the patient sinks during the stage of depression. When a surface either of great extent or high organization, has been burned, the patient dies soon after the injury, exhibiting symptoms merely of deranged and injured nervous function; the shock to the nervous system having been too violent to leave the capability of reaction. When power is left in the constitution sufficient to excite reaction, then some degree of hope remains for the patient; new symptoms, and a new source of danger less immediately imminent than the first, arise. Now this is precisely what happens in the most intense and dangerous forms of contagious fever; the most formidable cases being those, in which the power of bringing about reaction is taken away, and the patient sinks during the latent period.*" 491.

"* This is that variety of fever which Dr. Armstrong has designated congestive fever, a name derived from its supposed cause, viz. venous congestion: but those, who have seen the disease, or have read attentively his history of the symptoms, and who, at the same time, have observed other diseases of which an over-loaded state of the veins is a leading feature, will scarcely admit, that venous congestion (a change in the venous system, which, if it exist at all during life, is itself but an effect) can satisfactorily account for a series of symptoms, such as those which characterise this worst and most dangerous variety of fever."

Cholera morbus, especially the cholera of India, is instanced in illustration—many dying before re-action takes place.

In various diseases, the length of this latent period is various. Thus, in idiopathic fevers, it varies from a few hours to as many weeks or months:—In exanthematous fevers, its duration is more uniform. In certain other diseases, it endures to a length of time which is almost incredible, as, for example, in hydrophobia. Even in ague, the first paroxysm is very remote, sometimes, from the reception of the malaria. It is well known that the application of other noxious agents to the body will often accelerate the explosion of the disease. Thus, cold or intemperance will bring out the ague sooner than it otherwise would. It is also extremely probable, that the nature and force of the succeeding disease are greatly modified by accidents occurring during the latent period. A knowledge of this may be turned to a practical advantage.

"I am informed by Dr. Cheyne, to whom indeed I am indebted for this useful practical remark, that when symptoms of measles or other exanthematous fever appear in one member of a numerous family, it is his practice to put the other members upon their guard; he advises the diet to be regulated, and chills of cold, fatigue, and other exciting causes of disease, to be carefully avoided; that thus the impending fever may be rendered as safe and mild as possible; this is certainly useful advice, as the treatment of the patient, during the latent period, materially influences the progress and issue of the coming disease. Frequently we may observe in our hospitals cases, in which a debauch, excessive fatigue or exhaustion, exposure to wet or cold during the latent period, give to the fever a fatal character. From hence we learn one reason of the danger and fatality of fever amongst medical practitioners. During the latent period, with fever lurking in the system, they make an effort day after day to discharge their laborious duties; until at length they are reluctantly compelled to yield, the disease having gathered strength in the same proportion as they have made strong but ineffectual efforts to resist it." 496.

A minute investigation of the phenomena presented during the latent period of diseases, might probably afford important indications of the approaching storm. Thus, those who have slept in a damp bed often feel uneasy sensations in their joints for many days before the rheumatic fever sets in. Coryza in the eyes may lead us to expect measles, if the patient had been exposed to infection.

We may perceive that, in several of those cases which we have quoted at the beginning of this paper, one circumstance was very remarkable, namely, that many of the individuals were in a state of impaired health, debility, or exhaustion, at the time of exposure. This condition of the system would appear to be necessary in some cases to render the pestilential effluvia operative. The more the health is unimpaired and the system vigorous, the more will the morbific agency be resisted. "The nurses in hospitals are continually exposed to the poisonous effluvia, yet they sustain no injury, except they happen to be, at the time, exhausted or debilitated."

Dr. Marsh dwells at some length on the various circumstances which tend to render the body susceptible of the operation of febrific effluvia, as cold, intemperance, fatigue, the depressing passions—in short, the whole range of circumstances which derange any of the functions of body or mind. These are so well known that we need not dwell on the *mode* in which they produce the susceptibility to fever.

"From the foregoing considerations it will not be difficult to arrive at a knowledge of the means best calculated to guard against contagion. In the first place, those whose duty compels them to visit patients labouring under infectious diseases, should avoid, as far as is possible, the concentrated effluvia, which emanate from the persons of the sick; from the facts stated it appears that the poisonous particles arise from the excretions, and are mixed abundantly with the cuticular and pulmonary exhalations; hence it is not safe suddenly to throw back the bed coverings, or to be exposed to the breath of the patient, particularly when he coughs or expires fully. It is not safe to enter a small room in which the exhalations arising from the sick are confined, and accumulated; therefore an important safeguard is a free current of pure air. The power of conveying infection appears to encrease with the advance of the disease, and seems greatest at the commencement of convalescence. In fact all prudent means should be adopted to avoid exposure to a full dose of this virulent poison, which, when concentrated, is capable of acting injuriously, even upon those whose health is perfect, and whose strength is unimpaired; yet there are a few who seem to possess an inherent power of resisting the action of this as of other poisons, unless applied in such doses as to be inevitably fatal.—As to the distance at which it is asserted there is safety, I can say nothing, because I am not furnished with data whereon to ground an opinion. Since the danger of infection depends not merely upon the degree to which the air about the patient is impregnated with morbid matter, but also upon the *degree of health and strength* of the person exposed, it seems to me that precise and unvarying calculations on this subject are not to be relied upon. If the room be well ventilated, and the person who visits the sick in health and in full vigour of mind and body, he may, without incurring risk, approach the patient's bed, and remain near to his person. He should avoid, however, the effluvia confined under the bed clothes, and the breath of the patient labouring under typhus. To avoid exposure to the concentrated effluvia is the first precaution: the second I have to mention, and that not less in importance, is, to avoid exposure, even in a slight degree, at a time when the health is impaired, or when temporary debility or relaxation exist. Hence there is danger in exposure, immediately upon awaking from sleep, or at a moment of exhaustion from fatigue, from fasting, or from any other cause productive of debility. Being chilled with cold renders the frame susceptible of morbid impressions. An excess in eating or drinking, which rarely fails to be followed by exhaustion and debility, produces the same liability to receive disease, as insufficient and unwholesome nourishment. There are states of the atmosphere which produce, in a remarkable manner, this susceptibility of disease; this state of the air may, to a certain extent, be guarded against by attention to clothing, exercise, and diet." 517.

Dr. M. has met with some cases which incline him to believe that the *type* of fever has not any exclusive connexion with its cause—and that as much depends upon atmospheric influence or constitutional diathesis, as upon the immediate source of the disease. Thus malaria does not invariably produce intermittents, nor contagion continued fever.

Our author concludes with some remarks on the treatment suited to the stage of depression, or the latent period of fever. Unfortunately this is a period in which the physician is seldom summoned—hence the want of information on this important subject. It is of considerable moment in all cases, to investigate the *cause* of disease which has been applied. Thus, if it was an impression of cold upon the system, the means of counteracting the effects will be different from those employed where contagion has been received. In the latter case, our author believes, and we agree with him, that the fever can rarely be cut short. In general, and in spite of every effort, contagious disease will run a certain course—and, though it may be mitigated in severity, will seldom be stopped short in its career.

"The remedies which alone succeed in cutting short fever, are those which give a shock to the system; and the stronger the shock be, provided the constitution be able to endure its action, the more certainly will it succeed in extinguishing the disease. Occasionally, powerful emetics administered during the latent period, will give the system a shock sufficient to alter the course of the symptoms, and to enable the constitution to throw off the disease. Emetics, however, even at this early period, are not suited to all cases of fever. Sometimes I have known this remedy produce little other effect than determine morbid action to the stomach, and render that viscus exceedingly irritable during the whole course of the fever. When, therefore, it is the character of the existing fever to affect principally the stomach and intestines, emetics as also purgatives must be given with caution and reserve: and such emetic medicines should be selected as will not produce either too much nausea or too much debility. This is the reason that Ipecacuanha should in the majority of cases be preferred to Tartar emetic." 533.

The effects of an emetic will be rendered more effectual, if followed by an opiate. The abuse of purgatives, at the commencement of fever, is justly exposed by our author. No measure is perhaps of so much importance as absolute rest during the latent period. Every exertion, mental or corporeal, at this time, only increases the severity of the approaching fever. All exposure to wet or cold—all repletion in diet, ought to be carefully avoided. At this period, a change of air is highly advantageous, and mildly cordial and diluting drinks, keeping quietly in bed, would, we are convinced, nip many a fever and many other diseases in the bud.

Here we must close our analysis. Although Dr. Marsh has not been able to throw much new light on so obscure a subject as the latent period of fever, yet he has brought forward many interesting facts—especially those respecting contagion. It has, of late, been fashionable to doubt the agency of this poison as emanating from human living bodies—and to attribute all fevers to malaria. We think the facts, stated from such authentic sources by Dr. Marsh, will stagger the most sceptical. They will also be useful in putting young men, who have imbibed the new doctrines, on their guard, when they approach the beds of those who lie ill with the fevers in question.

